# HSV-1 ICP22 condensates impair host transcription by depleting promoter RNAPII Ser-2P occupation

**DOI:** 10.3389/fmicb.2025.1538737

**Published:** 2025-02-18

**Authors:** Hansong Qi, Mengqiu Yin, Xiaoli Ren, Guijun Chen, Ai Li, Yongxia Li, Xia Cao, Jumin Zhou

**Affiliations:** ^1^Key Laboratory of Genetic Evolution and Animal Models, Kunming Institute of Zoology, Chinese Academy of Sciences, Kunming, China; ^2^Kunming College of Life Science, University of Chinese Academy of Sciences, Beijing, China; ^3^Maize Research Institute, Sichuan Agricultural University, Chengdu, China; ^4^Department of Pulmonary and Critical Care Medicine, The Second Affiliated Hospital of Kunming Medical University, Kunming, China; ^5^Key Laboratory of Second Affiliated Hospital of Kunming Medical University, Kunming, China; ^6^KIZ/CUHK Joint Laboratory of Bioresources and Molecular Research in Common Diseases, Kunming, China

**Keywords:** HSV-1, ICP22 condensate, RNAPII, transcription, H3K27me3

## Abstract

Herpes simplex virus type I (HSV-1) infection-induced host transcript ion shutdown is one of the most critical hallmarks of viral lytic infection. However, how HSV-1 and which viral factors accomplish this dramatic effect is not well understood. In this study, we show that ICP22-defined condensates shutdown host global transcription but facilitate viral transcription. This is independent of its effects on viral infection-triggered changes in splicing, readthrough, and read-in events. ICP22 condensates depleted the serine-2 phosphorylated RNA polymerase II (RNAPII Ser-2P) occupancy from the host transcription start site (TSS), resulting in decreased host transcripts output. At the same time, it ensures proper RNAPII Ser-2P distribution on the viral genome to promote viral transcription. This effect is dependent solely on the condensate-forming activity, as condensate-disrupting point mutations abolish it. In addition, ectopic expressed ICP22 alone could decrease host transcription activity and increase histone H3K27me3 modification level. Thus, ICP22 condensates shut down host transcription by reducing RNAPII binding to host TSS to impair the host transcription.

## 1 Introduction

HSV-1 is an important pathogen with a high prevalence worldwide. Its productive, or lytic infection, is responsible for disorders including cold sores, keratoconjunctivitis, and the highly fatal herpes simplex encephalitis (Everett, 2014). The lytic infection of HSV-1 disrupts normal cellular functions and results in host transcription shutdown by the viral host shutoff (vhs) protein, which degrades both host and viral mRNAs (Friedel et al., 2021), and by infection-induced near-complete loss of RNA polymerase II (RNAPII) occupancy on the host genome (Abrisch et al., 2015). The cause for the latter effect—whether it is due to a cellular stress pathway or viral factors—is not known. Additionally, RNAPII promoter-proximal pausing, elongation and termination, and mRNA splicing are perturbed globally by HSV-1 infection—exhibited as promoter-proximal pausing peak extension (Birkenheuer and Baines, 2020; Weiss et al., 2023), elongation repression (Isa et al., 2021; Birkenheuer et al., 2022), disturbed splicing events (Hu et al., 2016; Tang et al., 2016), and readthrough transcripts (also called DoG) (Rutkowski et al., 2015; Wang et al., 2020; Djakovic et al., 2023).

These clues suggest that HSV-1 infection changes the host transcription at multiple levels to benefit viral replication. In particular, transcription readthrough, that is, RNAPII elongation passed through transcription termination sites, is mediated by viral ICP27, which blocks host transcription termination, and by viral ICP22, which reportedly repositions intergenic histone modifications (Wang et al., 2020; Djakovic et al., 2023). Readthrough events lead to the continuous elongation into the downstream gene region, producing the so-called read-in events. This type of transcriptional disruption is also seen in tumor cells and osmotic stressed cells (Mestdagh et al., 2010; Hangauer et al., 2013; Vilborg et al., 2015), although the biological effects have not been investigated.

The immediate early (IE) genes of HSV-1 are essential to viral reproduction and are best understood viral genes, except the IE gene ICP22. ICP22 deletion from wt virus led to a dramatic reduction of viral late gene transcription, lower viral yield, and virion quality (Orlando et al., 2006). ICP22 interacts with CDK9 and the facilitates chromatin transcription (FACT) complex, leading to the phosphorylation of RNAPII at serine-2 and viral transcription elongation (Durand et al., 2005; Zaborowska et al., 2014; Fox et al., 2017). However, our recent study (Qi et al., 2024) as well as other studies support the two different roles of ICP22 in regulating RNAPII Ser-2P levels, whereby it both induces and limits RNAPII Ser-2P degradation such that there is a loss of RNAPII Ser-2P occupation on host genome (Fraser and Rice, 2005, 2007; Zaborowska et al., 2014; Abrisch et al., 2015), but there is enough RNAPII for viral reproduction (Orlando et al., 2006; Mostafa and Davido, 2013). Supporting this, ICP22 is shown to work as the repressor of host transcription elongation and to reprogram the host epigenetic landscape to disrupt transcription termination (Isa et al., 2021; Djakovic et al., 2023). Moreover, overexpression of a conserved domain (193-256aa) in alphaherpesvirinae of ICP22 in cells decreased the RNAPII occupation on some host genes (Zaborowska et al., 2014).

Our recent study revealed that ICP22 forms condensates, and recruits RNAPII Ser-2P to prevent its degradation, and to ensure viral transcription; point mutations within a conserved domain in ICP22 disabled its phase separation activity, and the mutant virus mimicked the ICP22-null phenotype (Qi et al., 2024). We report that ICP22 condensates contributed to the HSV-1-infection-induced host transcription shutdown. These condensates are responsible for the loss of promoter RNAPII Ser-2P occupation. The presence of the condensates alone resulted in H3K27me3 modification alterations and shifted RNAPII Ser-2P from the host genome to the viral genome to benefit viral transcription.

## 2 Methods

### 2.1 Cell and virus

MRC5 (CCL-171) and Vero (CCL-81) cells were obtained from the American Type Culture Collection. 293 T (KCB 200744YJ) cells were obtained from the Conservation Genetics Academy of Science (CAS) Kunming Cell Bank. Cells were grown in Dulbecco’s modified Eagle’s medium (DMEM, Gibco) supplemented with 10% fetal bovine serum (FBS, Gibco), penicillin (100 U/mL), and streptomycin (100 μg/mL) in a humidified 5% CO_2_ atmosphere at 37°C. Herpes simplex virus type 1 17+ strain (NC_001806.2) was originally from Nigel Fraser. 17 + -ICP22-EYFP and 17 + -ICP22-Mut-EYFP were constructed and preserved by our lab. The virus was grown and titrated in Vero cells.

### 2.2 RNA-seq assay

MRC5 cells were seeded on the 6-well plate for 10^6^ each well for overnight culture, and distinct HSV-1 strain was separately inoculated to the cells as the multiplicity of infection (MOI) = 10, the mock-infection cells were added to the same volume of Vero cell culture medium. We have five groups, including mock, WT-6 h, WT-12 h, Mut-6 h, and Mut-12 h, with three independent replicates each. All the samples were collected at the indicated time points by adding Trizol to lyse cells, and the mock group’s samples were harvested at 12 hpi. The total RNA was extracted following the standard protocol, and the ployA-enriched RNA was used to construct the library and sequenced by the NovaSeq 6,000 (Novogene, China, Tianjin).

### 2.3 CUT&Tag assay

Approximately 10^5^ MRC5 cells were seeded into a 24-well plate for overnight culture, 12-h post wt and mutant virus infection (MOI = 10), mock-infection, and infected cells were digested by the 0.25% typsin, and the collected cells were centrifuged for 5 min (×500 *g*) to get the cell precipitate. The collected cells were used for nuclear extraction [steps within the TD903 kit (Vazyme, China, Nanjing)], and the following steps were guided by the manufacturer’s instructions (Hyperactive Universal CUT&Tag Assay Kit for Illumina [TD903, Vazyme, China, Nanjing]). Notably, 15-μl purified DNA went for library construction following the manufacturer’s instruction (TruePrep Index Kit V2 for Illumina [TD202, Vazyme, China, Nanjing]), and the PCR amplification cycle was 12. All the harvested DNA was sequenced by NovaSeq 6,000 (Novogene, China, Nanjing).

### 2.4 Western blot

Approximately 10^6^ MRC5 cells were seeded into a 6-well plate for overnight culture; wt and mutant virus were inoculated to the cells (MOI = 10), and the samples were harvested by radio-immune precipitation assay (RIPA) lysis solution in the indicated time points. The lysate was centrifuged for 10 min (12,000 rpm) to get the supernatant and boiled for 10 min, adding proper loading buffer. For the histone modification probing, 293 T cells were seeded into a 24-well plate for overnight culture and were transfected with 1-μg pEYFP-N1-ICP22 or pEYFP-N1-Mut-ICP22 (In-house) each well for 24 h; then, the samples were collected, following the method instructed by the manufacturer. All the Western blotting assays followed the standard protocol and the antibody usage, following the manufacturer’s instructions: RNAPII Ser-2P (1:2,000, Abcam, ab238146) (UK, Cambridge), *β*-tubulin (1:3,000, Abcam, ab179513) (UK, Cambridge), GFP (1:1,000, Invitrogen, MA5-15256) (USA, Rockford), ICP4 (1:1000, 58 s, in house, Gift of Gerd Maul) (In-house), rabbit polyclonal antibody for Histone 3, H3K4me3, H3K9me3, H3K27me3, and H3K27ac (1:500, in house, made by commercial company and preserved by our laboratory) (In-house kept polyclonal antibodies, originally made by Abclonal company, China, Wuhan); Goat Anti-Rabbit IgG (H + L) secondary antibody (31460, Invitrogen, USA), HRP (1:3,000, Invitrogen, 31,460, Invitrogen, USA), Goat Anti-Mouse IgG (H + L) secondary antibody (31430, Invitrogen, USA), HRP (1:3,000, Invitrogen, 31,430, Invitrogen, USA). The quantification of the band was performed on ImageJ (USA) (Schneider et al., 2012).

### 2.5 Real-time reverse transcription-polymerase chain reaction (qRT-PCR) assay

293 T cells were evenly seeded into 6-well plates for overnight culture, then transfected with pEYFP-N1-WT/Mut-ICP22 plasmid for 12 h. Harvesting the samples by treating cells with Trizol solution (RNAi plus, 9109, Takara, Japan) and extracting the total RNA by the standard protocol. The total RNA following rRNA erased was used to reverse transcription with the random primer. The cDNA was probed by the given primer pairs using the SYBR Green method (TSE201, TSINGKE, China, Beijing). The primers were as follows: MMP28-F: CAGGAGCTGCGCAAGGAG, MMP28-R: CACCCACTGAAACGCTCTGA; GALNT4-F: GGATTTCCCTGCATCGACAC, GALNT4-R: GTACGGAGCAAAGTCGACCA; SERPINB13-F: ACATCGATGGCCTGGAGAAG, SERPINB13-R: ATCGTAACCGTCCTCCACCT; GCNT2-F: CTGCCACGGCCACTATGTA, GCNT2-R: TCCACAGTAAGGGGGTAGGT; MAP2K6-F: AGCAGCTCAAACAGGTGGTA, MAP2K6-R: TCTGGGTATGTAGGCCGTTC; EBLN2-F: CCAACCCAGCATTGGGGATA, EBLN2-R: TGCCTGGTGTAACCCATCAA; RDH13-F: CGGCTTCGAGATGCAGTTTG, RDH13-R: AGTCTATGTGCCCAGCAACA; SLITRK5-F: GCGTCATTGAACCCAATGCT, SLITRK5-R: AGGTCCAAGTGCGTTAAGGG; CERS6-F: AGCCACTCACAACTGACCTT, CERS6-R: GGTGGTGCAGGAACATAATGC; GPR183-F: TGCAATGGGCTTTGACTGGA, GPR183-R: GGTGCACCACAGCAATGAAG; ZBED2-F: CTGCACTGTGGAAGCATCTG, ZBED2-R: GAGCCTACCCCAGTTACCCT; ARMCX5-F: TGGGTCCAGACCTGACAGAA, ARMCX5-R: CCAGAACCAGGACCCTACAC.

### 2.6 Bioinformatics

For RNA-seq data analysis, we trimmed the released raw by trim_galore (v0.6.7) with default parameter (specifically adding “--retain_unpaired,” owing to the HSV-1 originated transcripts that were mostly of high GC ratio, they cannot always get the precisely paired fragment), then aligned to the human reference genome hg38.p14 downloaded from the National Center for Biotechnology Information (NCBI) by hisat2 (v2.2.1) with default parameter (with “--un-conc-gz” parameter to get the HSV-1 originated reads for further alignment), while the unmapped reads were aligned to the HSV-1 reference genome NC001806 (GCA_000859985.2) by hisat2 with default parameters. SAM file converted to BAM file and its indexing completed by SAMtools (v1.16.1) (Li et al., 2009) and the BAM file to BW file (normalized by reads per kilobase per million mapped reads [RPKM]), the principal component analysis (PCA) analysis based on the BAM file, and the further genome-wide profiling were all accomplished by deepTools (v3.5.1) (Ramirez et al., 2016). All the read counts were quantified by featureCount (v2.0.1) (Liao et al., 2014) with default parameters. For CUT-Tag data analysis, the trimming adaptor was the same as above, while the bowtie2 (v2.5.0) (Langmead and Salzberg, 2012) were used to align the sequence to human reference genome hg38 by the “--sensitive-local” model with default parameters (with “--un-conc-gz” parameter to get the HSV-1 originated reads for further alignment), and the duplicated fragments were removed by sambamba (v0.8.2) (Tarasov et al., 2015) with default parameters (the HSV-1 reads alignment is the same). SMA and BAM file processing and the genome-wide profiling were the same as above (SAMtools; deepTools). Peak calling performed by macs2 (v2.2.7.1) (Zhang et al., 2008) with default parameters (without input of the control BAM file). ChIPseeker (Yu et al., 2015) was used for peak annotation. All further analyses based on the read counts, peak calling data, and figure generating were accomplished in R (v4.3.1). In detail, the differential analysis performed by DESeq2 (Love et al., 2014); the gene types annotated by biomaRt package; GO term annotated by clusterProfiler (Wu et al., 2021) and plotted by GOplot; the majority of figures were created by ggplot2.

### 2.7 Readthrough and read-in reads quantity

ARTDeco quantified all the readthrough and read-in transcripts (Roth et al., 2020) with the default parameters. The differential analysis of readthrough and read-in transcripts between wt, mutant, and mock outputted by the ARTDeco automatic flow. The raw count of readthrough and read-in transcripts from the ARTDeco output were used to evaluate its abundance related to the gene body counts, specifically, the readthrough or read-in counts were the input of the experimental group and the gene body counts of the same gene were the input of control group, which were analyzed by DESeq2 (*p* < 0.05). We defined the readthrough gene as the proportion of readthrough reads to gene body reads over 10% and the read-in gene as the proportion of read-in reads to gene body reads over 30%. We extracted the information of those genes from the GTF file of hg38.p14 for the profiling of virus-induced readthrough and read-in genes. Then, profiles the reads distribution of selected genes. Significantly, we overlapped the typical readthrough and read-in genes of wt- and mutant-infected cells, then selected the readthrough genes that appeared in both the wt and mutant group but did not belong to the read-in genes as the HSV-1 induced readthrough genes for profiling. In contrast, the overlapped read-in genes between wt and mutant groups were chosen as the HSV-1-induced read-in genes for profiling.

### 2.8 Splicing event analysis

Splicing events quantification was performed by rMATS (v4.1.2), and the output data were used for further analysis in R software. The rmats2sashimiplot was used for visualization of the splicing events. The gene types annotated by biomaRt (Durinck et al., 2009) package and the PCA analysis were performed by prcomp function in R software.

### 2.9 Statistics and data availability

All statistical analyses were performed in R4.3.1, and the sequencing raw data are available in the NCBI Sequence Read Archive (SRA) database (PRJNA1181924).

## 3 Results

### 3.1 ICP22 condensates regulate HSV-1 and host transcription

**Figure 1:**
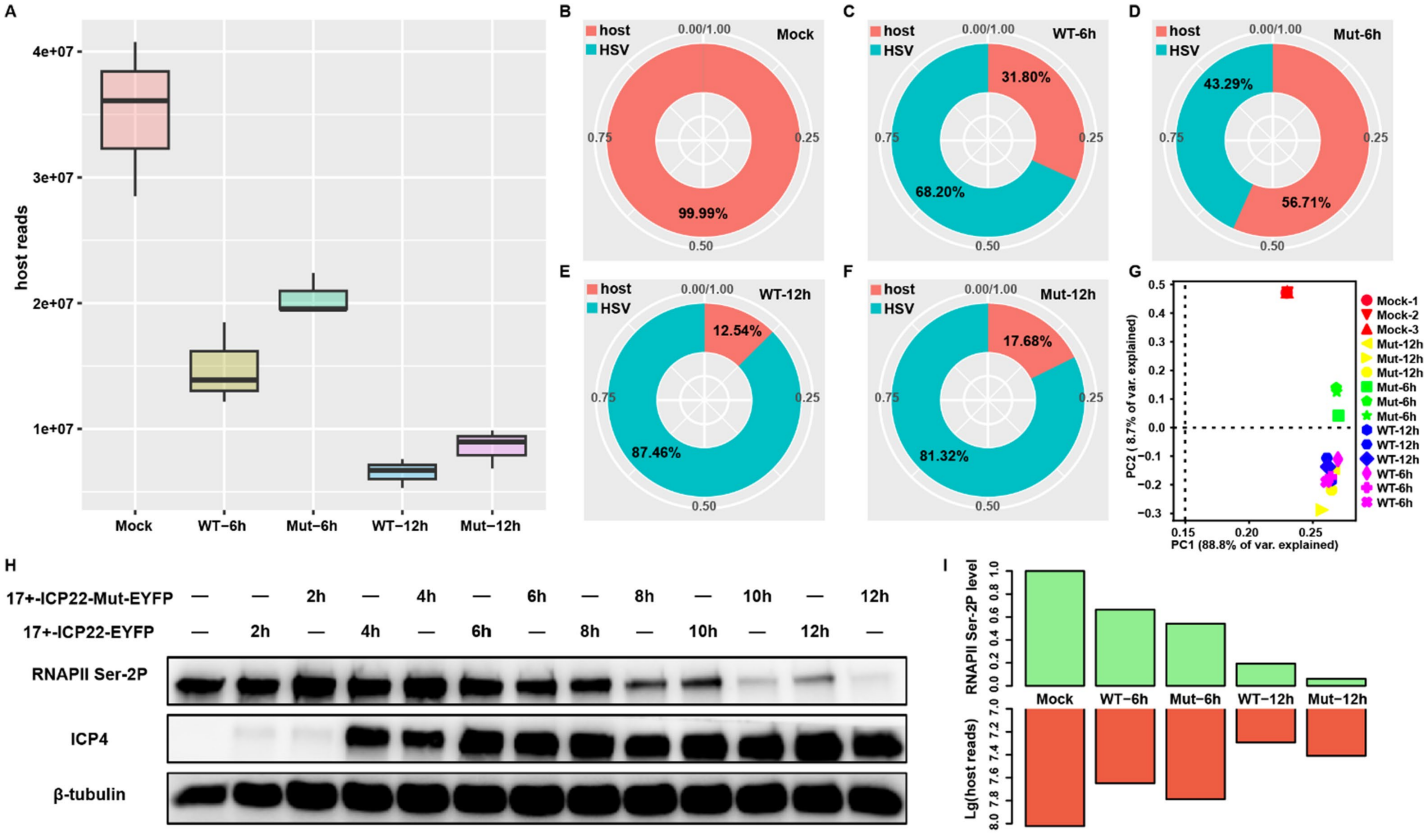
ICP22 condensates function in host transcription regulation. **(A)** The total mapped host reads of each group. **(B–F)** The ratio between host and viral reads among each group, the reads information extracted from the merged bam of each group. **(G)** The PCA plot is based on the BAM file of all samples. **(H)** The total RNAPII Ser-2P level of 2, 4, 6, 8, 10, 12 h post wt and mutant HSV-1 infection. **(I)** The quantified RNAPII Ser-2P level based on the Western blot corresponds to the mapped host reads quantity at 0, 6, and 12 h post wt and mutant HSV-1 infection.

To test the effects of ICP22 condensates on host transcription, we performed RNA-seq at 6 and 12 h post-infection (hpi) on wt (17 + -ICP22-EYFP) and ICP22 condensate mutant (17 + -ICP22-Mut-EYFP) HSV-1-infected MRC5 cells. As expected, the infection disrupts host transcription, as shown in decreased host reads in each infection group. However, the mutant virus-induced reduction of reads in the host was milder than that of the wt virus at both 6 and 12 hpi (Figure 1A). In detail, the viral reads of the wt group reached more than two thirds, at 68.20% of the total at 6 hpi, but the reads of the mutant group are less than half at 43.29%, which could be partly interpreted by the delayed viral life cycle of mutant HSV-1 (Figures 1C,D). The reads distribution discrepancy narrows between the wt and mutant-infected groups at extended infection (Figures 1E,F), where the total mRNA pool was predominantly occupied by the viral transcripts in both the wt and mutant virus-infected groups, at over 80% at 12 hpi. Nevertheless, the more abundant host transcripts of the mutant group at the late infection stage suggest that the disruption of ICP22 condensates affected host transcription. Indeed, host reads distribution based on PCA showed that the samples can be easily isolated into three clusters WT-6 h, 12 h, and Mut-12 h cluster, Mock cluster, and Mut-6 h cluster (Figure 1G). Intriguingly, the samples of WT-12 h are closer to WT-6 h than it is to the late infection stage samples of the mutant-infected group (Mut-12 h) (Figure 1G), indicating that the ICP22 condensates contributed to HSV-1-infection-induced host transcription disruption.

Our recent study showed that the disruption of ICP22 condensates caused nearly complete loss of RNAPII Ser-2P at late infection stage (Qi et al., 2024). In addition, a recent study points out that RNAPII pool size change regulates the transcription process (Tufegdzic Vidakovic et al., 2020). Thus, we wondered whether the RNAPII Ser-2P level change resulting from ICP22 condensates disruption impairs host transcription. To this end, we quantified the RNAPII Ser-2P levels of wt and mutant HSV-1-infected cells. We found that minor reductions of RNAPII Ser-2P level occurred at 6 hpi in the wt and mutant-infected cells, and that the reduction accelerated in extended infection (Figure 1H). Notably, the additional, severe RNAPII Ser-2P losses in mutant virus-infected cells were seen after 8 hpi. We, therefore, quantified the RNAPII Ser-2P levels at 6 and 12 hpi, and compared them to host RNA-seq reads, and found that the decreased RNAPII Ser-2P level led to an unexpected increase of the total host transcript as the infection progressed (Figure 1I). Specifically, the lower RNAPII Ser-2P level in the mutant virus-infected cells produced surprisingly more host transcripts at 6 and 12 hpi. This effect is more dramatic at 12 hpi, where the mutant virus-infected cells had a far greater amount of host transcripts than that of the wt-infected cells, even though the former has far less RNAPII Ser-2P protein than the latter (Figure 1I). These results strongly suggest that ICP22 condensates were disrupting host transcription.

### 3.2 ICP22 condensates contributed to the alteration of host transcription following HSV-1 infection

**Figure 2:**
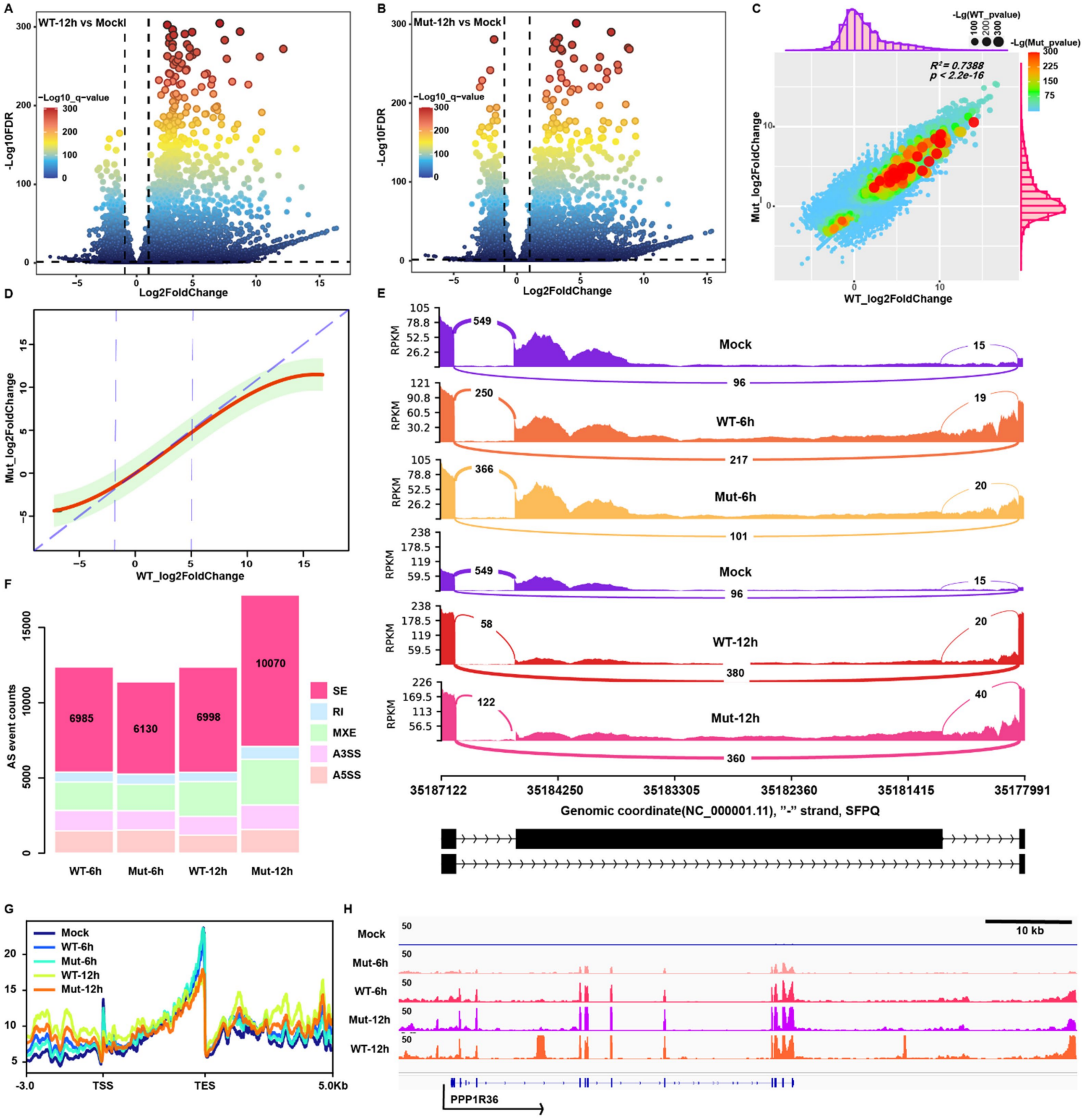
HSV-1-infection-induced transcription and splicing changes are independent of ICP22 condensates. **(A,B)** Volcano plot for the differential expression genes (DEGs) of wt and mutant infections at 12 hpi. **(C)** The correlation analysis between wt and mutant infections induces host transcription activity alteration based on the fold change information. **(D)** The linear regression model was constructed based on the fold change information of wt and mutant infection groups. **(E)** The representative splicing event (skip exon, SE) appeared on the SFPQ gene. **(F)** The distribution pattern of viral infection triggered splicing event changes. **(G)** Profiling the reads located in TSS-up 3 k to TES-down 5 k of each group. **(H)** Viral infection induced representative readthrough and read-in event on the PPP1R36 gene.

We performed differential expression analysis to profile the up- or downregulated genes in response to HSV-1 infection at 6 hpi in wt and mutant-infected MRC5 cells using bulk RNA-seq data (Supplementary Figures S1A,B). The wt virus infection induced a more extensive alteration of gene expression than that of the mutant at 6 hpi, likely due to the delayed life cycle of the mutant virus (Supplementary Figure S1C). To minimize this difference and measure how ICP22 condensates affect host transcription, we focused on the transcript at 12 hpi instead, when the total reads in the samples were dominantly taken over by viral transcripts. At this time point, there are large amounts of up- and downregulated genes (Figures 2A,B); however, the wt virus infection induced more drastic changes of gene expression than that of the mutant virus (Supplementary Figure S1D).

The majority of differentially expressed genes (DEGs) of these two groups overlapped, and the types of affected genes were also similar (Supplementary Figures S1E–G). Subsequently, linear regression analysis based on the differential fold change information showed a highly positive correlation with *R*^2^ = 0.7388 (Figure 2C). Remarkably, the linear regression function fitting indicates that wt virus infection triggers more severe changes of transcription activity in both up and downregulated genes than in the mutant (Figure 2D). Mutant HSV-1 infection shares similar alteration on transcriptome to wt infection, but was lower in magnitude compared to the wt infection.

### 3.3 ICP22 condensates did not affect alternative splicing during HSV-1 infection

We examined alternative splicing (AS) next to gain further insight into how the disruption of ICP22 condensates affects host transcription. Our previous study indicated that HSV-1 infection leads to extensive alterations to the host AS (Hu et al., 2016), and AS is also known to be hijacked by HSV-1 ICP27 protein to favor viral infection (Tang et al., 2016), but whether ICP22 condensates also play a role is not known. The five types of AS events induced by HSV-1 infection are shown (Figure 2E), highlighted by the skip exon (SE) events (Figure 2F). The SE-events-based PCA showed each sample’s diversity of SE event details (Supplementary Figure S1H). The AS event appeared to be independent of different stages of HSV-1 infection, as AS counts and distributions were undistinguishable between 6 and 12hpi. This holds for the ICP22 condensate-disrupting mutant, as both the wt and mutant showed similar AS event patterns at 6 hpi in their infected cells, respectively. At 12 hpi, the total and type-specific AS events in the mutant virus-infected cells surpassed that of the other groups. Still, the distribution patterns are similar, indicating that the disruption of ICP22 condensates did not prevent the rise of AS events after infection, and increased host transcription likely caused total AS event elevation. We thus conclude that the disruption of ICP22 condensates did not affect HSV-1 infection-induced alternative splicing.

### 3.4 ICP22 condensates did not contribute to HSV-1-infection-induced readthrough and read-in transcriptions

We profiled the mapped reads at transcription start sites (TSS) and transcription end sites (TES) to measure readthrough and read-in transcripts. As expected, HSV-1 infection halts global transcription but boosts the read-through and read-in transcripts, exhibited as decreased reads in the gene body, especially in the TSS and TES proximal region, and abnormally increased reads upstream of the TSS and downstream of the TES (Figures 2G,H).

**Figure 3:**
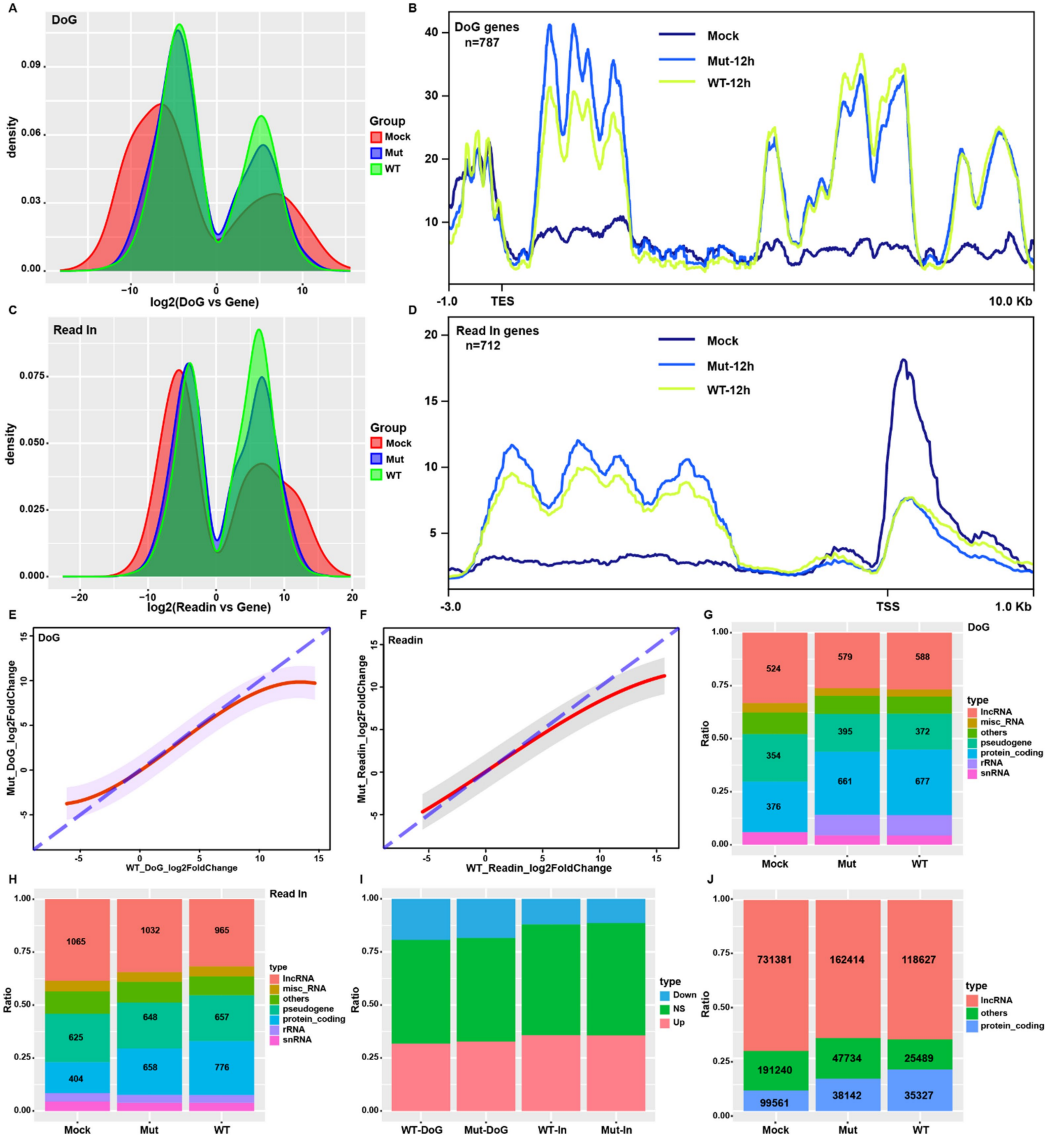
HSV-1-induced host readthrough and read-in transcriptions are independent of ICP22 condensates. **(A,C)** The distribution of the ratio of readthrough and read-in transcripts to gene body transcripts in each group (12 hpi samples for the viral group, same as above). **(B,D)** Reads profiling of the HSV-1-infection-induced readthrough and read-in genes. **(E,F)** Linear regression based on the wt and mutant infections induced readthrough and read-in transcripts fold change. **(G,H)** The gene type distribution of the readthrough and read-in genes for each group. **(I)** Overall distribution of up- and downregulated readthrough and read-in genes in wt and mutant-infected cells. **(J)** Proportion and quantity of lncRNA among the read-in transcripts for each group.

To analyze the contributions of ICP22 condensates, we quantified the HSV-1-infection-induced readthrough and read-in transcripts at 12 hpi by ARTDeco (Roth et al., 2020), and compared readthrough and read-in counts to the gene body counts by DESeq2. We obtained the following: First, readthrough and read-in events occurred in both normal and HSV-1-infected cells (Figures 3A,C), but HSV-1 infection changed the readthrough and read-in events distribution pattern, that is, readthrough and read-in transcripts appeared in some genes but lost in others. However, overall, the majority of the readthrough and read-in genes are shared among mock, wt, and mutant groups (Supplementary Figures S2A–C). Another characteristic is that HSV-1 infection narrows the readthrough and read-in transcripts distribution range and alters their peak locations, resulting in more readthrough and read-in transcripts. Here, the patterns are similar between wt and mutant virus-infected cells (Figures 3A,C). HSV-1 induced readthrough and read-in genes showed a dramatic increase of TES downstream and TSS upstream reads compared to controls (clean genes) (Figures 3B,D; Supplementary Figure S2B), suggesting that the infection increased genome-wide readthrough and read-in events (Figure 2G).

Further analysis revealed that the wt virus induced a more aggressive readthrough and read-in events than that of the mutant (Supplementary Figures S2D,F), shown as the stronger induced readthrough genes and induced read-in genes (Supplementary Figures S3E,F). However, the induction dynamic of readthrough and read-in by both the wt and mutant viruses are similar, and they mimicked the pattern shown in gene expression (Figure 2D), suggesting a direct linkage between readthrough and read-in products and gene transcription activity.

HSV-1 infection could perturb the established readthrough and read-in dynamic, but the analysis revealed that HSV-1 infection did not alter the distribution pattern of readthrough and read-in. At the same time, it elevated the ratio of that for protein-coding genes (Figures 3G,H). Linear regression analysis on readthrough and read-in fold changes uncovers the same kinetics between wt and mutant infections (Supplementary Figures S2E,G). Mutant and wt virus infection led to almost identical gene type, up and down trends for readthrough and read-in events (Figures 3G–I), suggesting that ICP22 condensates did not affect readthrough or read-in events.

It is of interest to note that lncRNA coding genes account for nearly 40% of total read-in genes and a third of total readthrough genes in normal cells (Figures 3G,H), suggesting that part of lncRNAs were produced by the readthrough events of upstream transcription so that the resulting read-in events to transcribe downstream lncRNA. More importantly, the lncRNA transcripts account for over 70% of total read-in reads in normal cells (Figure 3J), which supports that transcription read-in could be a possible way to express lncRNA under physiologic conditions.

### 3.5 ICP22 condensates perturb host transcription initiation

**Figure 4:**
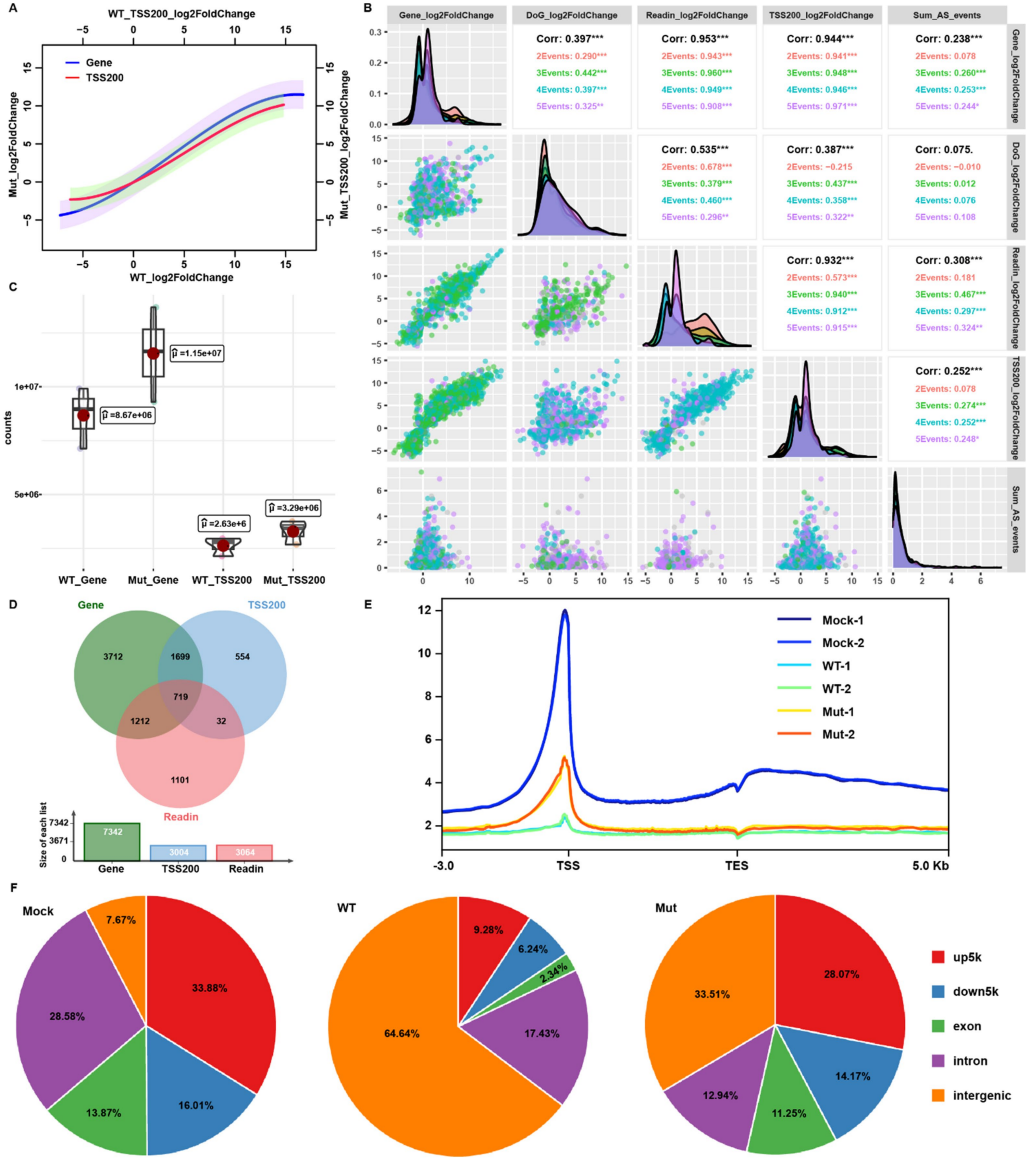
ICP22 condensates perturbed the host transcription initiation. **(A)** The linear regression based on the gene body or TSS200 reads fold change of wt and mutant groups. **(B)** Multiple correlation analysis between the gene expression, readthrough, read-in, TSS200 fold change, and splicing changes based on the wt 12 hpi samples’ data. **(C)** Total mapped read counts of gene body and TSS200 in wt and mutant-infected cells. **(D)** Venn diagram for the overlapping of significantly (|log2FoldChange| > 1, *p* < 0.05) changed gene, read-in, and TSS200. **(E)** RNAPII Ser-2P host genome occupation profiling ranged from TSS up 3 k to TES down 5 k. **(F)** RNAPII Ser-2P host genome occupation distribution related to the gene location.

Promoter-proximal RNAPII pausing and release is a critical transitional step from transcription initiation to elongation (Noe Gonzalez et al., 2021). A previous study reported that HSV-1 infection resulted in extended promoter-proximal pausing, and induced promoter-proximal peaks to even over 250 bp away from the TSS (Weiss et al., 2023). We extracted the reads from TSS to TSS + 200 bp (TSS200) to analyze the promoter-proximal pausing status to represent transcription initiation. TSS200 reads showed a similar trend to gene expression (Figure 4A), consistent with the generally accepted concept that promoter-proximal pausing controls transcription activity. Consistent with the larger number of mapped host reads of the mutant-virus-infected group over wt at 12 hpi (Figures 1A,E,F), the host gene TSS200 counts of the mutant-infected cells were all greater than that of the wt virus at 12 hpi (Figure 4C; Supplementary Figure S2H). Notably, mutant virus infection produced a higher amount of host transcripts, but lower viral transcripts compared to the wt virus infection at the same stage. This result suggests that the ICP22 condensates role in HSV-1 infection-triggered transcription shift from the host to viral genes to favor viral replication.

Since we have analyzed host transcription processes at the late stage of HSV-1 infection including transcription activity, alternative splicing, transcription-coupled readthrough and read-in events, and promoter-proximal pausing site reads occupancy, we performed multiple correlation analyses between gene expression fold change and each of the above-mentioned events at 12 hpi by the wt virus. The TSS200 reads occupation and read-in events correlated with gene expression status (corr = 0.953 and 0.932, respectively; Figure 4B). Interestingly, TSS200 reads occupation status also correlated to the read-in induction intensity, which could be attributed to the increase or decrease of upstream readthrough events (Figure 4B). We subsequently overlapped the genes with differentially occupied TSS200 reads and genes with read-in transcripts to the DEGs, and found that the majority of TSS200 reads occupation changed genes overlapped with DEGs. In contrast, read-in genes matched less well (Figure 4D). Read-in events were observed just in some of the genes, while TSS200 reads occupation change was a global observation; thus, promoter-proximal pausing governed TSS200 reads distribution could be the more reliable factor to interpret host transcription under HSV-1 infection.

Additionally, genes with differentially occupied TSS200 reads accounted for less than half of DEGs (Figure 4D), suggesting that gene body transcription output might be controlled by both initiation activity and RNAPII pausing (which produce abortive transcripts). Therefore, even though we detected the comparable TSS200 reads between the infected and mock group, many gene body reads would still be absent, following HSV-1 infection. Taken together, HSV-1-infection-induced global host transcription shutdown was due to both blocked transcription state transition and initiation, and likely by ICP22 condensates.

We further clustered genes based on the aforementioned five features by k-means algorithm to reveal how transcription changes due to infection (Supplementary Figure S2I). Gene ontology (GO) enrichment analysis (biological process [BP]) for each gene cluster showed that gene cluster 2, which showed induced gene expression and readthrough event (Supplementary Figure S2J), enriched in the transcription and mRNA splicing related process (Supplementary Figure S2K). Previous studies reported readthrough transcripts retained mainly in the nuclear, which will deprive the mRNA of cluster 2 genes from translation (Vilborg and Steitz, 2017; Rosa-Mercado and Steitz, 2022). This intriguing observation reminds us that HSV-1-infection-induced transcription shutdown is complex, but this highly orchestrated process deserves a closer look.

### 3.6 ICP22 condensates are responsible for HSV-1-infection-induced transcription shift from the host to the viral genome

To explore the roles of ICP22 condensates in the transcriptional shift from host to the viral genome, we conducted CUT&Tag assay to probe RNAPII Ser-2P occupation in both wt and condensate disrupting mutant HSV-1-infected cells at 12 hpi. Genome-wide RNAPII Ser-2P occupation profiling uncovers that HSV-1 infection triggered a dramatic global RNAPII Ser-2P occupancy loss (Figure 4E), which explains host transcription shutdown. Notably, the mutant induced a mild loss of RNAPII Ser-2P occupation at the TSS than that of the wt (Figure 4E). Quantifications based on the position of peaks relative to genes indicate that there is a dramatic redistribution of RNAPII binding. In uninfected cells, over 92% of RNAPII interacted with protein-coding genes, but in the wt virus-infected cells, over 60% peaks translocated to the intergenic positions, and TSS upstream occupation declined from 34% to less than 10%. When cells were infected by the mutant virus, the similar shift was observed, but to a much lesser degree. This is especially the case for the 5 k region located upstream of the TSS (Figure 4F).

**Figure 5:**
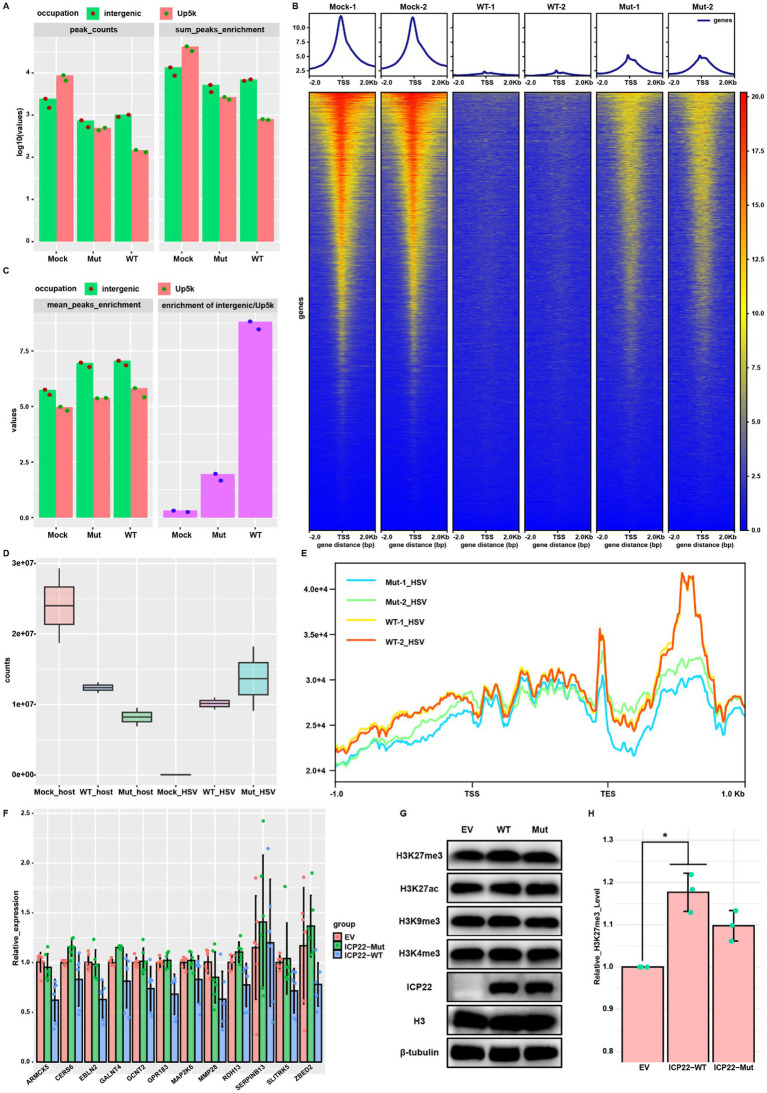
ICP22 condensates shifted transcription from the host to the viral genome. **(A)** Intergenic and TSS up 5 k occupied reads quantity (left) and total reads enrichment (right). **(B)** Heatmap for the peaks enrichment range from TSS up 2 k to TSS down 2 k. **(C)** The mean reads enrichment located in intergenic and TSS up 5 k (left), and the ratio between intergenic reads and TSS up 5 k reads (right). **(D)** Total mapped reads occupied on the host and viral genome of each group. **(E)** RNAPII Ser-2P viral genome occupation profiling ranged from TSS up 1 k to TES down 1 k. **(F)** RT-qPCR probed gene expression of the selected ones at 12 h post wt and mutant ICP22 coding plasmid transfection to 293 T cells. **(G)** Quantification of the selected histone modification level by Western blot at 12 h post wt and mutant ICP22 coding plasmid transfection to 293 T cells. **(H)** Quantification of the histone H3K9me3 modification at 12 h post wt and mutant ICP22 coding plasmid transfection to 293 T cells, based on three independent Western blot results.

Consistently, we quantified the total number of RNAPII peaks (peak_counts) located in TSS up 5 k and intergenic and their overall and mean enrichment fold (sum/mean_peaks_enrichment) and found HSV-1 infection reduced the TSS up 5 k enrichment, but elevated intergenic enrichment, while mutant infection showed a similar trend, but to a much lesser degree (Figures 5A,C, left). Infection by the wt virus strongly increased the ratio of intergenic binding over TSS up 5 k occupation than that of the mutant, and both are higher than the mock infection group (Figure 5C, right). Similarly, reads profiling around TSS showed the almost depleted RNAPII signal and disrupted distribution pattern in wt virus-infected cells. However, in mutant-infected cells, there is more resemblance to uninfected cells, although with a significantly decreased signal intensity (Figure 5B). These results demonstrate that ICP22 condensates are responsible for the depletion of RNAPII from host gene promoters.

Next, we checked the mapped reads on the host and viral genome. Taking into account the much lower RNAPII Ser-2P protein level in mutant-infected cells than that of the wt at 12 hpi, the overall RNAPII Ser-2P occupancy in the host genome of mutant-infected cells was expectedly weaker than that of the wt (Figure 5D), but the TSS proximal peaks in the mutant group were far more enriched than that in the wt group (Figure 5B), which strongly suggests that ICP22 condensates are responsible for HSV-1-infection induced loss of RNAPII Ser-2P at the host TSS region. To our surprise, the mapped reads in the viral genome showed the opposite trend (Figure 5D), with fewer reads in the TSS upstream and TES downstream region on the viral genome in mutant-infected cells (Figure 5E), which indicates that RNAPII could not be adequately positioned to efficiently transcribe viral genes when ICP22 condensates were disrupted (Figures 5D,E). Taken together, we conclude that ICP22 condensates depleted RNAPII Ser-2P from the host gene TSS region, and were essential to promote proper RNAPII Ser-2P distribution on the viral genome.

We therefore tested ICP22 condensates in the absence of viral infection by transfecting either the wild type or mutant ICP22 coding plasmid into 293 T cells for 12 h, then probed 12 genes that were downregulated by viral infection. As expected, the expression of these genes was also decreased by ectopic expressed wild type ICP22, even though the decrease is milder than that did the wt virus infection (Figure 5F). In contrast, ectopically expressed mutant ICP22 barely perturbed the expression of these genes (Figure 5F). This result confirmed the vital roles of the integral ICP22 condensates for host transcription disruption.

We also tested whether the presence of ICP22 condensates affects the level and modifications of histones and found that the presence of ICP22 condensates specifically increased the level of histone H3K27me3, while H3K9me3, H3K27ac, and H3K4me3 were unaffected (Figures 5G,H). This result suggests that ICP22 could directly or indirectly increase the number of repressive histones to silence host genes and promote the viral life cycle.

## 4 Discussion

HSV-1-infection-induced host transcription shutdown has long been reported, but which viral factor(s) is driving this process and how it is achieved is still not well understood. Here, we demonstrated that ICP22 condensates depleted RNAPII Ser-2P from the host TSS, resulting in the failure of transcription initiation, and consequently shutting off host transcripts. However, viral transcription was not impaired. The phase separation property of ICP22 is essential for this shift from host transcription to viral transcription. This represents a new type of activity where by the virus hijacks the host transcription machinery.

HSV-1 infection also affected splicing, RNAPII readthrough and read-in, but these effects are independent of ICP22 condensates. Instead, they were tightly linked to the infection state, i.e., the changes at 6 hpi were milder than that at 12 hpi. Interestingly, host transcription activity was highly correlated with readthrough and read-in occurrence. These two events also appeared in the normal cells, and the balance of the readthrough dynamic could be broken by disruption of cellular homeostasis including osmotic stress and oncogenesis (Mestdagh et al., 2010; Vilborg et al., 2015).

Although the function of readthrough in a normal cell is elusive, our data provide a potential linkage between readthrough transcripts and lncRNA. The majority of read-in reads mapped to the lncRNA coding genes, which could be transcribed by the readthrough events of protein-coding genes. Consequently, the cellular stimulations perturbing protein-coding gene transcription could affect the downstream lncRNA expression, which could potentially regulate the lncRNA. In addition, the readthrough transcripts were retained in the nucleus, as they could not be transported to the cytoplasm (Vilborg and Steitz, 2017; Rosa-Mercado and Steitz, 2022). This could quickly switch on or off genes in urgent situations. Thus, readthrough events have the potential to regulate many biological processes, and HSV-1 likely hijacked this mechanism to benefit viral reproduction.

When ICP22 condensates were disrupted, more than half of RNAPII Ser-2P TSS proximal occupation was restored on the host genome. Still, there was decreased binding to the viral genome, specifically upstream of TSS and downstream of TES. A likely mechanism could be that ICP22 recruits RNAPII Ser-2P away from the host genes, into ICP22 condensates, from there RNAPII was transferred to the HSV-1 replication compartment, likely with the help of other viral factors and loaded onto the viral TSSes (Durand et al., 2005; Bastian et al., 2010). It has been reported that the mediator complex also forms condensates, which selectively recruit RNAPII based on the C-terminal domain (CTD) phosphorylation state, resulting in precisely controlled transcription (Guo et al., 2019). The promoter-proximal pausing of RNAPII is governed by the NELF, which also forms condensates, and is regulated by the positive transcription elongation factor b (P-TEFb) to phosphorylate NELFA, and RNAPII then disperses the NELF condensate and facilitate RNAPII elongation (Bergeron-Sandoval and Michnick, 2018; Guo et al., 2019; Rawat et al., 2021; Richter et al., 2022). Our data showed that ICP22 condensates may compete with the host-induced condensates at the transcription initiation stage to block the elongation. Of note, a similar modus operandi has been employed by herpes tegument protein ORF52 to compete with cGAS to interact with DNA for forming ORF52-DNA condensates, thus repressing the innate immunity activation (Xu et al., 2021). Furthermore, ICP22 condensates could alone change the global H3K27me3 histone modification without viral infection, and ICP22 drives the gene downstream histone modification reposition (Djakovic et al., 2023). These evidence suggest that ICP22, and likely in cooperation with other viral factors, alters the epigenetic landscape following viral infection to repress host transcription. This essential function, together with the RNAPII processive role of ICP22 condensates, makes ICP22 a crucial therapeutic target against diseases due to HSV-1 infection.

## 5 Conclusion

HSV-1 infection suppresses host transcription and promotes viral transcription via ICP22-mediated condensate formation. These condensates diminished the occupancy of serine-2 phosphorylated RNA polymerase II (RNAPII Ser-2P) at host TSSs, thereby reducing host transcriptional output while ensuring the appropriate distribution of RNAPII Ser-2P on the viral genome to facilitate viral transcription. Furthermore, ectopic-expressed ICP22 decreased cellular transcription and elevated histone H3K27me3 modification. Those findings provide a deeper understanding of virus-host interaction at the transcription level.

## Data Availability

The datasets presented in this study can be found in online repositories. The names of the repository/repositories and accession number(s) can be found at: https://www.ncbi.nlm.nih.gov/, bioproject/PRJNA1181924.
