# Supplementary material for: HSV-1 ICP22 condensates impair host transcription by depleting promoter RNAPII Ser-2P occupation
Source: Front Microbiol. 2025 Feb 18;16:1538737. doi: 10.3389/fmicb.2025.1538737 (PMC11876393; doi:10.3389/fmicb.2025.1538737)
Supplement: Supplementary file 1 [file Data_Sheet_1.pdf]

Supplementary Material

1 Supplementary Figures

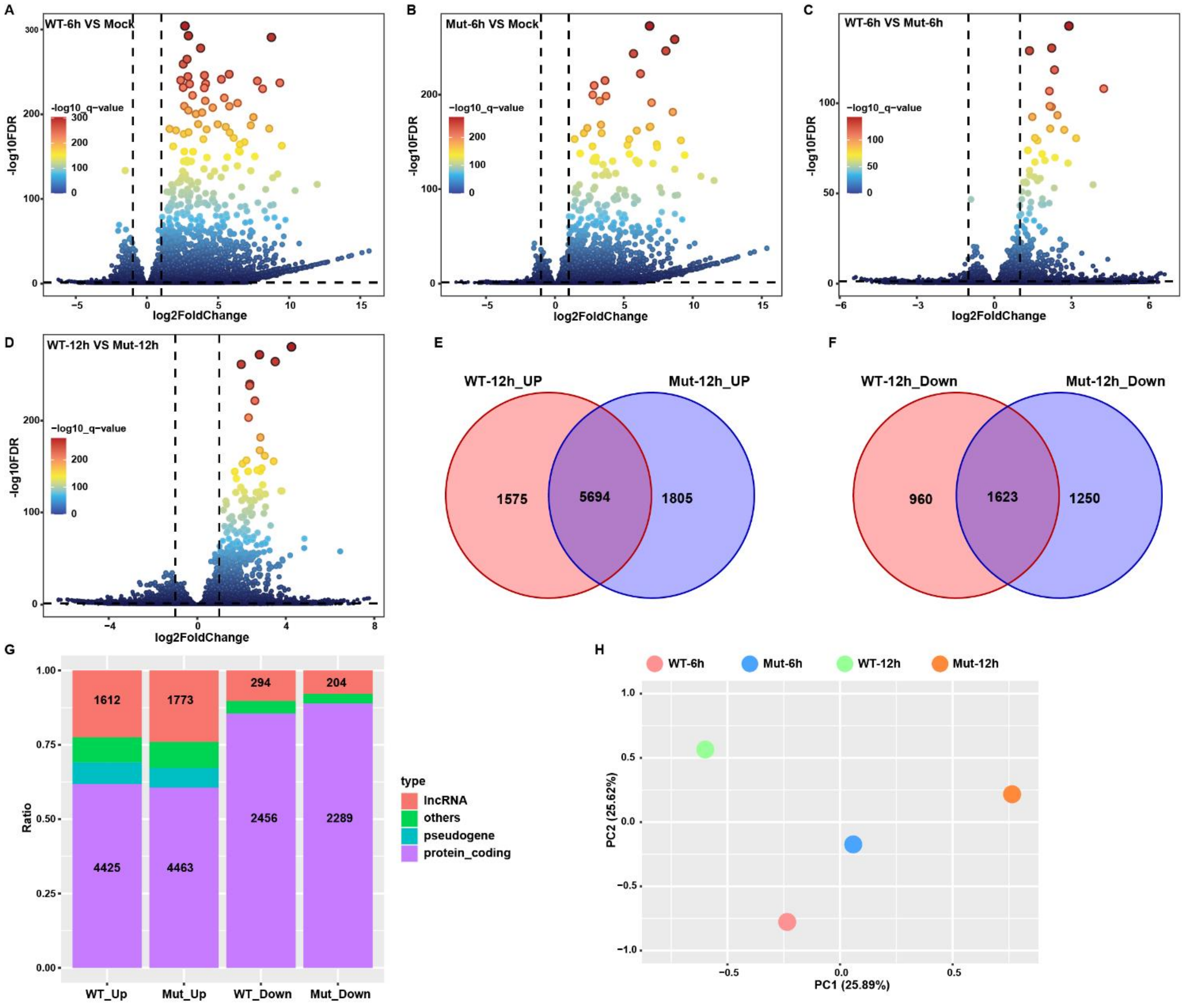

Supplementary Figure 1. Mutant and wt HSV-1 infection induced the comparable host transcription activity alteration and splicing events generating. (A-D) Volcano plotting for wt and mutant viral infection caused DEGs, including WT-6h (A) and Mut-6h (B) compare to mock group, WT-6h (C), 12h (D) compare to Mut-6h,12h separately. (E-F) Overlapped up and down regulated genes between mutant and wt HSV-1 infected cells at12 hpi. (G) The up and down regulated gene type distribution

of mutant and wt infection group at 12 hpi. (H) PCA plot based on the skip exon splicing events information of each infected groups.

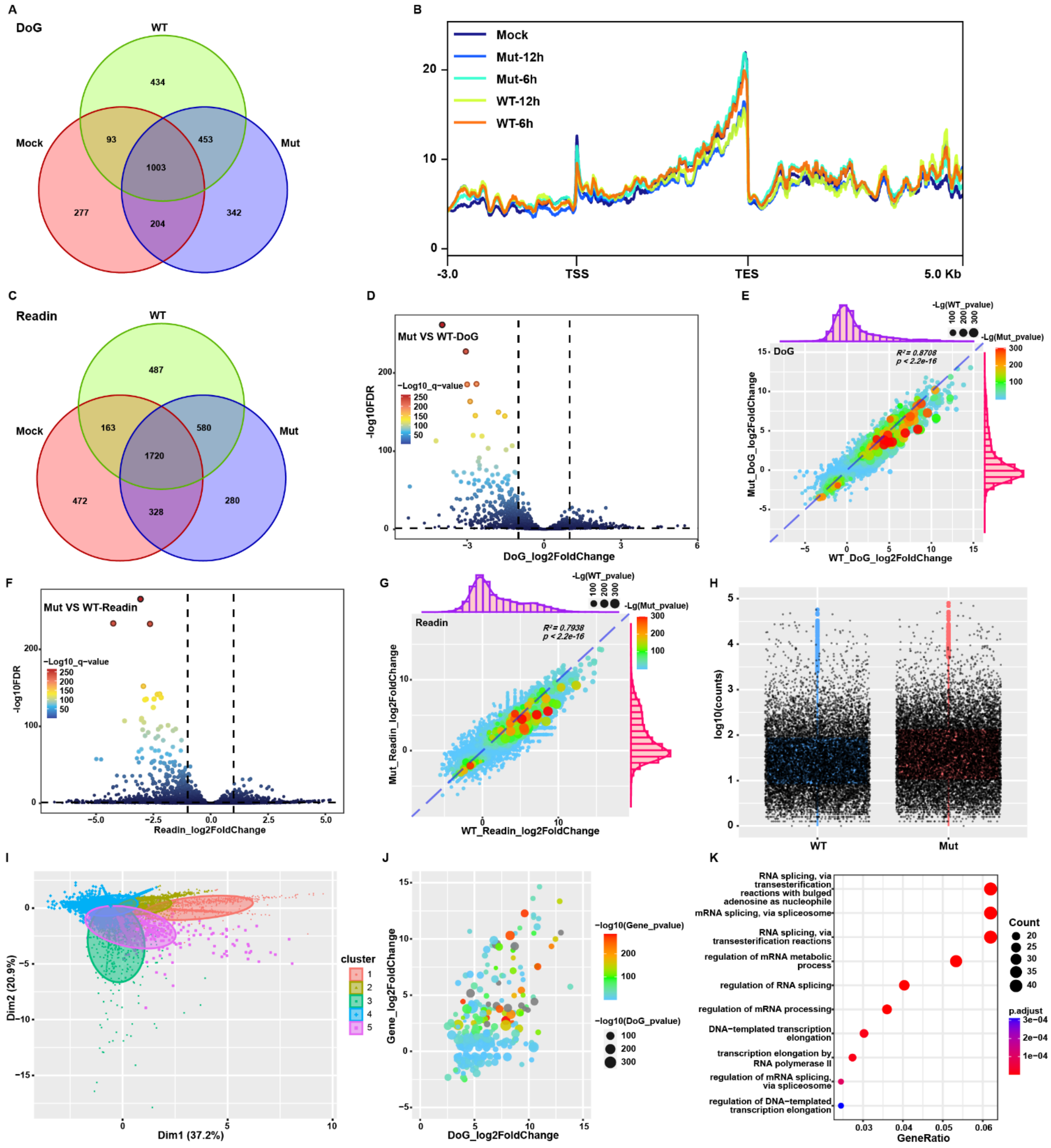

Supplementary Figure 2. HSV-1 infection induced readthrough and read in events is independent of ICP22 integrity. (A and C) Overlapped readthrough and read in genes between mock, wt and mutant

group. (B) Profiling the clean (both non-readthrough and read in genes) genes' reads located in TSS up 3k to TES down 5k of each group. (D and F) The differential expressed readthrough and read in genes between wt and mutant infected cells at 12 hpi. (E and G) Readthrough and read in transcripts fold change based correlation analysis between wt and mutant group at 12 hpi. (H) TSS200 reads count distribution of each single gene for wt and mutant infected cells. (I) Clustered genes based on the five-dimension data by k-means method, including gene expression-, read-through-, read-in-, TSS200- fold change and splicing changes. (J) Gene expression and readthrough fold change distribution of cluster 2 genes. (K) GO enrichment of cluster 2 genes.
